# On the Application of Entropy Measures with Sliding Window for Intrusion Detection in Automotive In-Vehicle Networks

**DOI:** 10.3390/e22091044

**Published:** 2020-09-18

**Authors:** Gianmarco Baldini

**Affiliations:** European Commission, Joint Research Centre, 21027 Ispra, Italy; gianmarco.baldini@ec.europa.eu; Tel.: +39-0332-78-6618

**Keywords:** Controller Area Network (CAN), cybersecurity, information entropy, in-vehicle network, Intrusion Detection System (IDS)

## Abstract

The evolution of modern automobiles to higher levels of connectivity and automatism has also increased the need to focus on the mitigation of potential cybersecurity risks. Researchers have proven in recent years that attacks on in-vehicle networks of automotive vehicles are possible and the research community has investigated various cybersecurity mitigation techniques and intrusion detection systems which can be adopted in the automotive sector. In comparison to conventional intrusion detection systems in large fixed networks and ICT infrastructures in general, in-vehicle systems have limited computing capabilities and other constraints related to data transfer and the management of cryptographic systems. In addition, it is important that attacks are detected in a short time-frame as cybersecurity attacks in vehicles can lead to safety hazards. This paper proposes an approach for intrusion detection of cybersecurity attacks in in-vehicle networks, which takes in consideration the constraints listed above. The approach is based on the application of an information entropy-based method based on a sliding window, which is quite efficient from time point of view, it does not require the implementation of complex cryptographic systems and it still provides a very high detection accuracy. Different entropy measures are used in the evaluation: Shannon Entropy, Renyi Entropy, Sample Entropy, Approximate Entropy, Permutation Entropy, Dispersion and Fuzzy Entropy. This paper evaluates the impact of the different hyperparameters present in the definition of entropy measures on a very large public data set of CAN-bus traffic with millions of CAN-bus messages with four different types of attacks: Denial of Service, Fuzzy Attack and two spoofing attacks related to RPM and Gear information. The sliding window approach in combination with entropy measures can detect attacks in a time-efficient way and with great accuracy for specific choices of the hyperparameters and entropy measures.

## 1. Introduction

With the evolution of the automotive industry to increased levels of connectivity and automation, the potential for cybersecurity attacks is growing as the vehicle is more exposed to digital attacks. A modern vehicle today is implemented with various electronic components including sensors, actuators, Electronic Control Unit (ECU) and communication devices, which are connected to different types of in-vehicle networks. The use of sensors to perceive the surrounding environment (e.g., camera, LiDARs) will be even more important with increasing levels of automation. In general, each ECU in a vehicle performs a specific function and groups of ECUs are usually connected into a common sub-network (e.g., powertrain). One of the most common in-vehicle network standards in the automotive industry is the Controller Area Network - bus (CAN-bus), which has such a widespread use due to its characteristics of low cost, relatively high reliability and fault tolerance. On the other hand, the CAN-bus was designed in times where the potential for cybersecurity threats was minimal due to the physical isolation of the vehicle from the outside world. Although tampering of specific functions was reported, this was usually implemented on specific components of the vehicle rather than the in-vehicle network. Because the automotive vehicles are increasingly connected, the potential for cybersecurity attacks grows from the technical point of view. Researchers have demonstrated the feasibility of various remote attacks starting with the seminal work by Checkoway described in [1], which has shown that the remote exploitation of a vehicle is possible via a broad range of attack vectors to the point that remote control of the vehicle can be achieved. Although the drivers for such attacks can vary and it may be related or not related to infringements of automotive regulations, the research community has started to investigate in-vehicle cybersecurity threats and potential countermeasures in detail. Although the connectivity of the vehicle and its dependency on quite sophisticated digital components does widen the risk of cybersecurity threats, the growing levels of automation of the vehicle increase the potential impact of a cybersecurity threat from a safety point of view as the driver may be absent or s(he) may not be able to react in due time. In the ICT domain, one of the primary techniques to mitigate cybersecurity threats is Intrusion Detection System (IDS) where the objective is to detect an attack in the shortest time possible so that an appropriate countermeasure (e.g., isolation of a section of an in-vehicle network) can be implemented. As mentioned above, the most important assets in the automotive vehicles are ECU, sensors and actuators, which are connected through various in-vehicle networks like CAN-bus, FlexRay and LIN. Then, a remote attack is likely to be conducted by injecting or manipulating messages in the in-vehicle network and this is the area where most of the research literature has focused (see Related Work Section 2). The advantage of IDS based on the analysis of in-vehicle traffic is that it does not rely on the implementation of cryptographic solutions in the in-vehicle network, which may be expensive to deploy or unfeasible to implement in existing standards or technologies because of technical limitations [2,3]. In this paper, we focus specifically on attacks on the CAN-bus, at it is the most widely deployed in-vehicle network standard in the world and it is mainly used to connect the most critical assets (e.g., ECUs) of the automotive vehicle. This paper proposes an approach based on the application of an information entropy-based method based on a sliding window, which is quite efficient from time point of view, it can be flexible to adapt to changes in the operational context of the vehicle and it provides a very high detection accuracy as demonstrated by the results presented in this paper. The approach is based on the calculation of the entropy of the CAN-bus messages transmitted on the CAN-bus network and it is based on the hypothesis that attacks modify the entropy of the CAN-bus traffic so that variation of the calculated entropy may indicate a cybersecurity threat. This idea is not new in the literature and recent studies have demonstrated its potential in comparison to other IDS techniques based on machine learning and deep learning (mostly from a time efficiency point of view), but in some cases the entropy-based approach has provided a low detection accuracy or the analysis of the attacks was limited to one or two cases. This paper proposes an extensive analysis of a wide variety of entropy measures, which explains the reason for the weak results presented in the literature (e.g., the selected entropy measures did not have significant discriminating power) and support the identification of the entropy measures, which provide the highest classification performance. Different entropy measures are used in the evaluation: Shannon Entropy, Renyi Entropy, Sample Entropy, Approximate Entropy, Permutation Entropy, Dispersion and Fuzzy Entropy. The paper evaluates the impact of the different hyperparameters present in the definition of entropy measures on a very large public data set of CAN-bus traffic with millions of CAN-bus messages with four different types of attacks: Denial of Service, Fuzzy Attack and two spoofing attacks related to RPM and Gear information. The sliding window approach in combination with entropy measures can detect attacks in a time-efficient way and with great accuracy for specific choices of the hyperparameters and entropy measures

This paper is organized as follows: Section 2 provides a state of art of the related work in two main areas: (a) intrusion detection systems in in-vehicle networks with a specific focus on the application of entropy measures and (b) application of the entropy measures used in this paper in various domains for problems of identification and classification. Section 3 describes the overall methodology for intrusion detection including the definition of the entropy measures used in the analysis and the materials (i.e., public data set for in-vehicle attacks) on which the measures are applied. Section 4 provides the results of the evaluation for the different attacks and the different entropy measures in relation to the values of the hyperparameters and the size of the sliding window. Section 5 draws conclusions and describes future developments.

## 2. Related Work

As mentioned before, this related work section focuses on two different areas: (a) intrusion detection systems in in-vehicle networks with a specific focus on the application of entropy measures and (b) the application of the entropy measures used in this paper for identification problems in other domains.

### 2.1. Intrusion Detection in In-Vehicle Networks

The basic principle of an IDS for an in-vehicle detection system is the same as that of an IDS for general communication networks, where the research literature is already quite extensive and in many cases, research outputs have led to commercial developments. There are already surveys in the literature, which extensively identify and catalog the different IDS for in-vehicle networks [4,5,6]. One simplistic classification of the methods to design and implement the IDS divides the literature in two main categories. The first category of methods is based on the pre-storing of pre-specified signatures of external attacks, inspection of the transmitted packets, and analyzing whether any pattern matches with the stored signatures. In this context, machine learning approaches based on the creation of a training data set of known attacks and normal/legitimate traffic are widely used and some examples can be found in [7], which uses one-class Support Vector Machine (SVM) or deep learning approaches as in [8]. Although these methods have achieved very high accuracy, they suffer from high computational costs to create the training set of the library of signature/patterns. The second method detects abnormalities using statistical characteristics of the normal range of the data generated by the components in the vehicle and transmitted in the in-vehicle network. This approach is more time-efficient than the approaches in the previous category because the extraction of the features from the network traffic lead to a significant dimensionality reduction in the data analysis process. On the other side, this method can be less accurate if the features and the related detectors are not chosen properly. The second method (adopted in this paper) can benefit from the specific characteristics of in-vehicle automotive networks. As highlighted in [9], one of the most relevant differences between conventional communication networks and vehicular networks in the viewpoint of IDS is that messages generated and transmitted in in-vehicle networks have uniform and regular characteristics, because the traffic usually conveys control or status information of the vehicle, unlike those made by network users over general networks. Because the estimation is made by determining whether the abnormal phenomenon is normally deviated from the specific pattern of normal traffic, the probability of error can be reduced, compared to general communication networks. It should also be noted that the computational power of the ECUs used in vehicles is generally limited compared to the processing power of the computing platforms in generic communications networks (e.g., Software Defined Networks), and thus, the implementation and execution of complicated classification algorithms (e.g., Deep Learning) may be difficult to implement in the vehicle. For this reason, this paper proposes an approach belonging to the second category where the detection of the in-vehicle attacks is done using features (i.e., the entropy measures in this case) extracted from the in-vehicle network traffic. It is noted that there are other approaches for IDS (as described in [4]) based on the physical layer fingerprinting of the ECU in the network to identify masquerade attacks when a malicious ECU replaces a legitimate one [10,11], but these techniques are out of scope of the approach proposed in this paper.

One of the first papers to adopt an entropy-based approach for in-vehicle detection is [12]. Although this can be considered a seminal paper in this area, their experimental evaluation in [12] was limited, and spans over just 15 s of CAN-bus traffic including only a single class of CAN-bus messages that are not safety-relevant.

This paper is mostly based on the approach proposed in [3,13], but the scope of this paper is considerable wider than each of the two papers. The first paper in the literature, which used entropy to detect in-vehicle attacks was [3], where the authors have used Shannon Entropy to detect two types of attacks: a replay attack and a fuzzy attack. A sliding window where the entropy is calculated and evaluated against a threshold k is used and a similar approach is used in this paper. This initial study was based on the timing of the messages and the detection accuracy suffered when the rate of attacks is relatively low. It was demonstrated in subsequent papers [13] (see paragraph below for further details) that an approach based on the counting of the messages is more effective than the timing of the messages. For this reason, this paper uses the number of received CAN-bus messages, while maintaining the information theoretic approach based on entropy measures.

In addition to [3], the most similar paper in the literature to this paper is [13], where a sliding window approach is used to detect two different types of attack: a DoS Attack and an injection attack. The impact of different window types is evaluated as well as the threshold used to determine when an attack is implemented or not. In comparison to [3] where a time-based approach was used, the authors in [13] use the number of received CAN-bus messages, which is shown to be more effective than the time-based approach. This paper benefits greatly from the description of the approach presented in [13] as it basically adopts a similar methodology but this paper expands considerably the analysis in [13] as it evaluates four attacks and uses a much wider set of entropy measures in comparison to the single use of Shannon Entropy. In addition, this paper uses a public data set, which is much larger than the one used in [13] (about 3.5 million CAN-bus messages). The data analysis is dependent on the choice of various hyperparameters, which include the choice of the entropy measures, the size of the sliding window and the threshold to distinguish a statistical anomaly (i.e., which points to an attack) from the previous calculated normal range. The last two hyperparameters are optimized using a simulated annealing algorithm. Instead, this paper opts for a description of the results according to the variation of the hyperparameter values to provide a more transparent view of the impact of each hyperparameter on the classification performance.

A very recent paper, which adopts a different entropy measure from the previous papers is [9] where the Renyi Entropy of order 2, 3 and 4 is used to detect a DoS and Fuzzy attack for different values of the sliding window. This paper is also based on a similar methodology to [9], but it takes in consideration a much larger set of entropy measures including Renyi Entropy.

Another significant difference of this paper in comparison to the previous references [3,9,13] is that the analysis is performed on the payload rather than the CAN-ID. This approach is proposed both to address a gap in the literature (mostly focused on the analysis of the CAN-ID) and to evaluate if the information theory approach can be applied to payload analysis and to address the threat where an attacker masquerades the injected traffic using a legitimate ID already present in the in-vehicle network. Although it is acknowledged that different vehicle models may have different semantics of the payload data (but they must be conforming to the CAN-bus standard specifications), the approach proposed in this paper is agnostic to the payload data semantics as it is based on a data analysis of the in-vehicle CAN-bus traffic and it needs to be executed only on a specific vehicle model or even a specific vehicle. See also Section 2.3 for additional details.

### 2.2. On the Application of Entropy Measures to Classification and Identification Problems

Most of the entropy measures used in this paper have never been used for in-vehicle attacks and there is an absence of related literature. On the other side, entropy measures are often used for identification problems in other domains. Thus, this subsection reviews the literature on the application of the entropy measures used in this paper (e.g., Sample Entropy, Approximate Entropy, Permutation Entropy, Dispersion Entropy and Fuzzy Entropy) for the purpose of detection and identification in different domains. Please note that the definitions of the different entropy measures are only briefly introduced in this section as they are described in detail in Section 3.5 of this paper. Permutation Entropy (PeEn) was initially introduced by Bandt and Pompe in [14] and it has been used for many different applications since then. In [15], it has been used for the detection of stealthy attacks on industrial control systems. The approach is based on the consideration that stealthy attacks present some sort of regularity besides the magnitudes, which prompted the adoption of PeEn in this paper as well because industrial control systems and in-vehicle networks shares some similarities. In the biomedical sector, PeEn is used in [16] to distinguish between normal and pathological gait with very good accuracy.

Sample Entropy (SaEn) and Approximate Entropy (ApEn) has been extensively used in the analysis of physiological signals, where it has often demonstrated superior performance. Even if there are many papers in the literature using SaEn and ApEn, we select the two following works since they are similar to our approach as they compare the discriminating power of different entropy measures for classification purposes. The authors in [17] have used approximate entropy with other entropy measures for the identification of focal electroencephalogram signals. The entropy measures are applied to the intrinsic mode functions generated by the application of empirical mode decomposition, while in this paper, the Fourier Transform is used. SaEn and other entropy measures have also been used in automatic sleep classification [18].

Dispersion Entropy (DiEn) has been recently introduced by the authors in [19] and it is suggested as an improvement both to PeEn and SaEn. Since its introduction, it has been applied for identification problem in different domains. In particular, for the identification and authentication of wireless communication devices, DiEn has demonstrated an improvement in the classification performance and robustness in the presence of noise [20]. DiEn has also been used to detect and identify gear faults in mechanical related applications, where it has shown its superior performance in comparison to PeEn and ApEn with the additional advantage of a faster computational time [21].

Fuzzy Entropy has been used in [22] in combination with the empirical Wavelet Transform for the monitor and diagnose of faults of motor bearing, where it has demonstrated its high identification performance. Fuzzy Entropy has been applied in combination with ant colony optimization in [23] to a problem similar to the one presented in this paper: intrusion detection in communication networks, which have different characteristics than in-vehicle network traffic.

In the same context of intrusion detection in communication networks, Renyi Entropy has been compared to Shannon Entropy in [24,25] to classify the traffic as normal or suspicious and to select the most appropriate attributes of the network traffic. Renyi Entropy has demonstrated a superior detection performance, which is the reason it was included in the set of entropy measures used in this paper.

### 2.3. Main Contributions of This Paper in Comparison to Related Work

To summarize, the key aspects of the proposed approach are identified in the following list:

- This paper extends significantly the number and types of entropy measures used (Shannon, Renyi, Sample, Approximate, Permutation, Dispersion and Fuzzy Entropy) to perform the information theory analysis in comparison to the limited number of entropy measures adopted in the literature. Some of these entropy measures (e.g., dispersion entropy) were introduced only recently in the literature in different domains than automotive cybersecurity. The rational for their use is that such entropy measures have demonstrated their discriminating power in classification problems and this paper evaluates their application to this specific domain. In addition the impact of specific hyperparameters (e.g., embedding dimension) present in the definition of some of the entropy measures is evaluated.
- Four different type of attacks (identified as DoS, Fuzzy, RPM and Gear in the rest of this paper) are analyzed in comparison to the literature on a published data set containing millions of CAN-bus-messages.
- The analysis is performed on the CAN-bus message payload rather than the CAN-bus message ID as commonly done in the literature because the ID could subject to masquerading attacks. As highlighted in [26], the analysis of the payload rather than the CAN-bus IDs presents the issue that a large amount of data must be processed, especially if machine learning of deep learning approaches are used. This is the reason an efficient sliding window approach is instead used in this paper where a large set of entropy measures is applied to reduce the dimensionality of the CAN-bus payload data. It can be remarked that different vehicle manufacturers have different semantics of the payload content in the CAN-bus messages, but the objective of this paper is not to support portability of the attack detection approach across different vehicle manufacturers. The intrusion detection system can be specific to each vehicle or to a vehicle model where the payload format and semantic is the same. Then the payload-based IDS is based on the consideration that the IDS algorithm identifies the key values of the hyperparameters using a data derived approach and it is agnostic to the implementation/format of the CAN-bus payload in the vehicle model.

## 3. Materials and Methods

### 3.1. Description of the Controller Area Network Protocol

CAN-bus protocol was invented by Robert Bosch GmbH and officially released in 1991. It is a message-based protocol, which was designed to allow robust communication among microcontrollers in a vehicle and meet the specific requirements of in-vehicle environment, such as real-time processing, strong robustness, and cost effectiveness. CAN-bus protocol uses broadcast communication to transmit messages.

**Figure 1:**
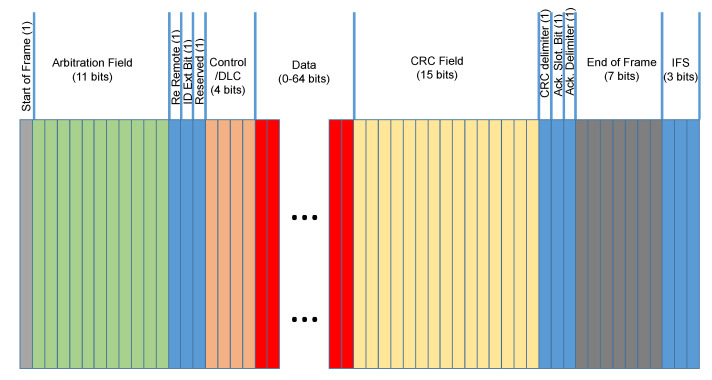
Standard CAN-bus message frame.

A description of the standard CAN-bus (CAN 2.0) frame structure with the identification of the specific fields is provided in Figure 1.

As mentioned before, the focus of this paper is on the analysis of the Data segment of the frame, which can be composed up to 8 bytes. Because of the increasing data exchange in in-vehicle networks, in most of the cases, all the 8 bytes are used and the data set used in this paper (described in Section 3.2) has all the CAN-bus messages set to 8 bytes. As described before, it can be remarked that different vehicle manufacturers have different semantics of the payload content in the CAN-bus messages, but the objective of this paper is not to support portability of the attack detection approach across different vehicle manufacturers. The intrusion detection system can be specific to each vehicle or to a vehicle model where the payload format and semantic is the same.

### 3.2. Data Sets and Attack Scenarios

This paper uses the data set created by Hacking and Countermeasures Research Lab described in [26,27]. The data has been extracted from a Hyunday YF Sonata through a Y-cable plugged into the OBD-II port through a Raspberry Pi3 as described in [26,27]. The recorded CAN-bus traffic matches the specification of CAN 2.0 with a CAN-bus message interpretation based on the Hyunday YF Sonata model.

The datasets contain each 300 intrusions of message injection. Each intrusion performed for in time ranging from 3 to 5 s, and each dataset has total 30 to 40 min of the CAN-bus traffic, then the data sets are quite extensive and they contains millions of messages as described in the following Table 1:

The four attacks scenarios are described below:

- In the Denial of Service (DoS) attack, messages of ‘0000’ CAN-bus ID were inserted in the in-vehicle network every 0.3 ms.
- In the Fuzzy attack, totally random CAN-bus ID and DATA values of the CAN-bus message were injected every 0.5 ms.
- In the Spoofing attack of type RPM, messages related to the RPM information were injected every 1 ms.
- In the Spoofing attack of type Gear, messages related to the Gear information were injected every 1 ms.

The Dataset were created by logging CAN-bus traffic (from 30 to 40 min of CAN-bus traffic) via the OBD-II port from a real vehicle while message injection attacks were performing. Additional details are provided in [26,27].

### 3.3. Workflow

The description of the workflow for the processing of the data is described in this section. As described in the introduction, a sliding window approach is implemented where a set of CAN-bus messages is used to generate a sample for the data analysis process. The number of CAN-bus messages used to create the sample is defined by the parameter $W_{s}$ in the rest of this paper. $W_{s}$ is the window size used for the analysis. For example, a window value of $W_{s}=24$ generates a sample of size 192 bytes because each CAN-bus message in the data set has a data payload of 8 bytes. On the basis of the results from literature [13], which provides an indication of the suitable range of window size, we identified four different values of the window size $W_{s}$: 24,72,120 and 168. The trade-off is that a larger $W_{s}$ may decrease the reaction time while a smaller window size may provide a lower detection accuracy. These assumptions are evaluated in Section 4. In a similar way to what it has been done in the literature, a sample of size $W_{s}$ is considered normal/legitimate (Note: the terms legitimate traffic and normal traffic have the same meaning in the rest of this paper.) if the sample contains only normal CAN-bus messages. The sample is considered malicious (e.g., an attack is being implemented) if it contains even a single CAN-bus message labeled as malicious in the data set. The use of a moving window allows a faster detection of the attack as the CAN-bus messages are processed in ‘batches’ (i.e., the samples) rather than a single CAN-bus message at the time. In this paper, the choice is to avoid overlapping among samples: no CAN-bus message belongs to two samples at the same time. The reason for this choice is to foster time efficiency as overlap would obviously increase the detection time. On the other side, the proposed approach can be easily extended to overlapping samples, where the percentage of overlapping become an additional hyperparameter in the analysis.

There are three main phases in the application of the approach proposed in this paper: the normal traffic estimate, the training phase and the operational detection phase. This paper is mainly focused on the normal traffic estimate and the training phase, but the evaluation of the detection phase is also performed. The evaluation of the classification performance of the different hyperparameters is quite similar to the workflow presented in [13] with the difference that the analysis is performed on the payload rather than the CAN-ID and no meta-heuristics algorithms are used to identify the optimal hyperparameters as it is preferred to present the graphs showing the impact of each hyperparameter and leave the choice of the optimal values to the IDS designer. Each phase and the related steps are described in the following paragraphs.

**Figure 2:**
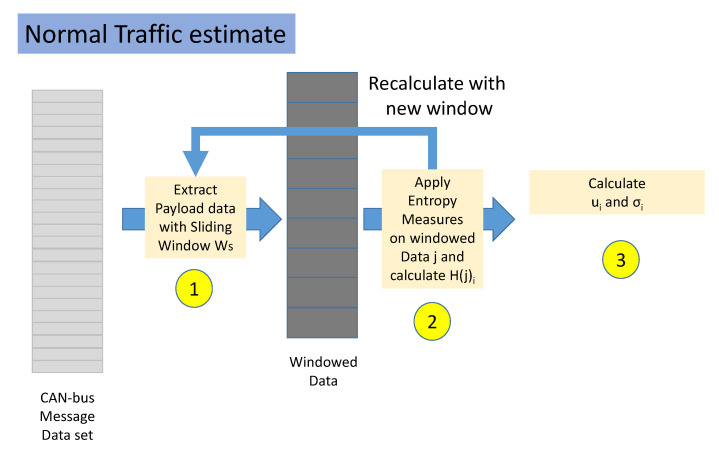
Estimate of the normal traffic entropy values.

The workflow of the normal traffic estimate is described in Figure 2. This phase is executed only on data labeled as normal. The workflow is composed by the following steps.

- **Step 1**. The normal traffic portion of the data set is split in non-overlapping windows. Each window is composed by several CAN-bus messages equal to $W_{s}$.
- **Step 2**. For each window, the value of each Entropy Measure $H{\left(j\right)}_{i}$ is calculated where *j* is the identifier of the window and *i* is the identifier of the Entropy Measure. This step is repeated until all the data set has been analyzed.
- **Step 3**. For each Entropy Measure *i*, the mean $u_{i}$ and standard deviation $\sigma_{i}$ is extracted from all the values of H calculated in the previous step.

**Figure 3:**
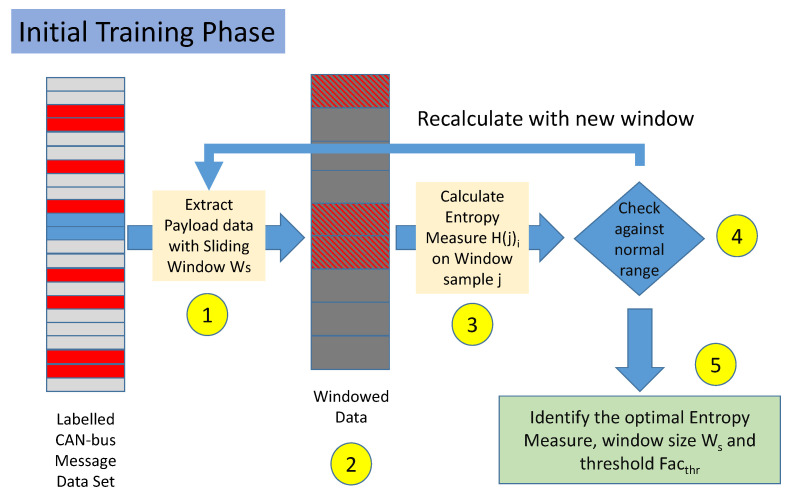
Training phase and hyperparameters optimization.

The training phase is described in Figure 3 and it is composed by the following steps:

- **Step 1**. The labeled data set is split in non-overlapping windows. Each window is composed by several messages equal to $W_{s}$. In the rest of this paper, the set of $W_{s}$ messages is also called a sample.
- **Step 2**. Each sample is labeled as malicious if it contains at least a CAN-bus message, which was initially labeled as malicious. If all the messages are labeled as legitimate, then the sample is labeled as legitimate.
- **Step 3**. For each sample j and each Entropy Measure i, the value $H{\left(j\right)}_{i}$ is calculated.
- **Step 4**. For each sample *j* and each Entropy Measure *i*, the value of $H{\left(j\right)}_{i}$ is compared against the mean $u_{i}$ and standard deviation $\sigma_{i}$. If the difference between $H{\left(j\right)}_{i}$ and $u_{i}$ in absolute value is less than a threshold, the sample is predicted as legitimate, otherwise, it is considered malicious. These conditions are formally defined in Equations (1) and (2) below in Section 3.4. Steps 3 and 4 are repeated for all the samples in the data set.
- **Step 5**. Steps 1–5 are repeated for different values $W_{s}$ and for different values of the threshold $Fac_{thr}$.

**Figure 4:**
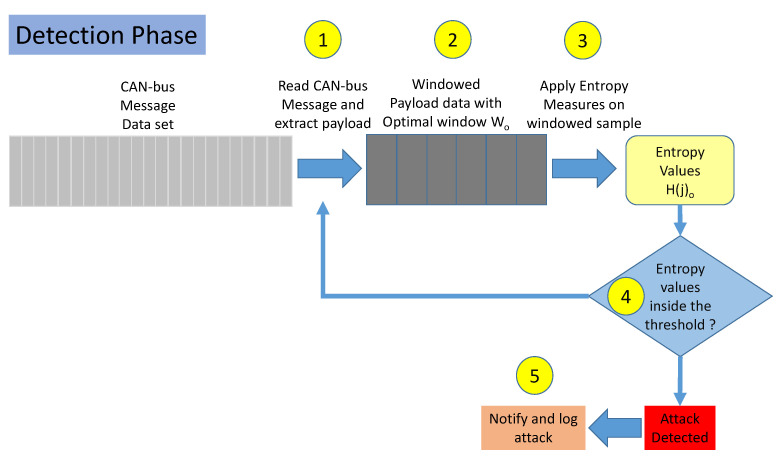
Detection phase of the traffic.

The operational Detection phase is described in Figure 4. It is based on the previous phases as it is the phase where the IDS in the vehicle monitors the in-vehicle network traffic to detect attacks. This phase is composed by the following steps:

1. **Step 1**. The payload data is extracted from the CAN-bus message data set from a set of sequential messages.
2. **Step 2**. Samples are generated by collecting a set of $W_{o}$ CAN-bus messages. $W_{o}$ is the optimized window size.
3. **Step 3**. The entropy measures identified as optimal are used to calculate $H{\left(j\right)}_{o}$ from each sample *j*.
4. **Step 4**. It is checked if $H{\left(j\right)}_{o}$ is within the range defined by the optimized threshold $Fac{\left(o\right)}_{thr}$ as described in Section 3.4.
5. **Step 5**. If the previous step 4 shows that $H{\left(j\right)}_{o}$ is out of the threshold range, an attack is reported and logged.

A significant design choice is related to the portion of the data set, which are used to estimate the mean and the standard deviation (i.e., the Normal Traffic Estimate described in Figure 2) against the training phase where the optimal values of the hyperparameters are calculated (i.e., described in Figure 3). Although other papers in the literature identify a specific ratio (e.g., half for training and half for testing), this paper evaluates the impact of the size of the training and test set, which is expressed with the parameter $R_{TT}$, which is defined as the ratio of the portion of the data set used for Normal Traffic Estimate against the overall data set (in this case, it is used only the traffic labeled as normal). Then, the training/hyperparameters evaluation and the detection phase is performed using the remaining $\left(1-R_{TT}\right)$ fraction of the entire data set. The potential trade-off (to be evaluated in the Results Section 4) is that a larger training set requires more training time but it may lead to a more accurate detection of the malicious traffic.

### 3.4. Performance Metrics

The performance metrics to detect an attack are similar to what is used in the literature: Accuracy, Precision and Recall related to a binary classification problem between legitimate traffic and attacks. Then, a True Positive (TP) is when a traffic sample (i.e., set of CAN-bus messages in a window of size $W_{s}$) is predicted by the algorithm as legitimate and it is true that it is legitimate. A False Positive (FP) is when a traffic sample is predicted to be legitimate, but it is actually malicious (i.e., part of an attack). A False Negative (FN) is when a traffic sample is predicted to be malicious, but it is actually legitimate. Finally, True Negative (TN) is when a traffic sample is predicted to be malicious and it is indeed malicious.

A traffic sample of size $W_{s}$ is considered normal/legitimate or malicious respectively on the condition defined in the following equations:

(1) $${\mathrm{Normal}⟼|H\left(j\right)}_{i}-u_{i}|<Fac_{thr}\times \sigma_{i}$$

(2) $${\mathrm{Malicious}⟼|H\left(j\right)}_{i}-u_{i}|>Fac_{thr}\times \sigma_{i}$$

where *i* is the identifier of the Entropy Measure and $Fac_{thr}$ is one of the hyperparameters to define the threshold factor, which discriminate between normal and malicious traffic.

The main goal is to maximize the number of correctly predicted traffic samples on the overall data set, which leads to the definition of accuracy as:

(3) $$Accuracy=\frac{\left(TP+TN\right)}{\left(TP+TN+FP+FN\right)}$$

Another goal is to minimize the number of FP and FN (or maximize their inverse) and in particular FP as it is more dangerous that the algorithm wrongly predicts legitimate samples as malicious than the reverse. This leads to the other two metrics used in this paper:

(4) $$Precision=\frac{\left(TP\right)}{\left(TP+FP\right)}$$

(5) $$Recall=\frac{\left(TP\right)}{\left(TP+FN\right)}$$

These metrics are used to evaluate the proposed algorithm in relation to the variation of the various hyperparameters described in the previous sections and subsections: $R_{TT}$, $W_{s}$, $Fac_{thr}$, type of attack and the parameters defined for the entropy measures.

### 3.5. Entropy Measures

This section describes the entropy measures, which are adopted for the analysis presented in this paper. Beyond the classical or textbook definition of the entropy measures, the focus of this section is to identify the key hyperparameters in the definition of the entropy measure, which could impact the detection of the attack. In addition, in some cases, there are constraints on the length of the time series on which the entropy measure must be applied, which are discussed in detail in Section 3.13.

### 3.6. Shannon Entropy

(6) $$ShEn=-\sum_{i}^{N}p\left(x_{i}\right)log\left(p\left(x_{i}\right)\right)$$

where $p(x_{i})$ is the probability $p(x=x_{i})$.

For reproducibility of the results presents in this paper, the entropy MATLAB function from MATHWORKS was used.

### 3.7. Renyi Entropy

(7) $$ReEn=\frac{1}{1-\alpha }log\left(\sum_{i}^{N}p{\left(x_{i}\right)}^{\alpha }\right)$$

where $p(x_{i})$ is the probability $p(x=x_{i})$. The limit for α⟶1 is the Shannon Entropy defined above. In this paper, we adopt the values of α=2,3,4 as these are the range of values used in the literature [9].

For reproducibility of the results presents in this paper, the MATLAB implementation of the Renyi Entropy available at [28] was used.

### 3.8. Permutation Entropy

PeEn was introduced by Bandt and Pompe in their seminal paper [14]. The concept is to define an entropy measure, which takes into account the time causality of the time series (causal coarse-grained methodology) by comparing neighboring values in a time series. Then, PeEn is the Shannon Entropy of a sequence of ordinal patterns—the latter being discrete symbols that encode how consecutive time series entries relate to one another in terms of position and value and it is defined by the following equation:

(8) $$PeEn=-\sum_{i}^{N!}p_{i}^{{}^{\prime }}log\left(p_{i}^{i}\right)$$

where $p_{i}^{{}^{\prime }}$ represents the relative frequencies of the possible patterns of symbol sequences, termed permutations. The permutation is related to a sequence of *m* (embedding dimension) values of the original series. A time delay τ can be used in the generation of the permutations from the original series, but for simplicity we set the value of τ=1 in this paper, while the value of *m* is an hyperparameter to be optimized. Additional details on the definition of the PeEn are provided in [14].

For reproducibility of the results presented in this paper, the MATLAB implementation of the Permutation Entropy provided by the authors in [29] was used in this paper.

### 3.9. Dispersion Entropy

DiEn was recently introduced in [21] and it addresses the potential weakness of PeEn where the mean value of amplitudes and differences between amplitude values are not considered in its definition. In dispersion entropy, the initial series $X=x_{i},x_{i+1},\ldots ,x_{N}$ is mapped to *c* classes. Although this mapping can be implemented with various linear or non-linear approaches, the authors in [21] propose to use Normal Cumulative Distribution Function (NCDF) to map × to the *c* classes. Then, the implementation of the DiEN is similar to PeEn, with the generation of dispersion patterns rather than permutations and with the calculation of the probabilities $p(\pi_{j})$ on the basis of an embedding dimension *m* and the time delay τ. As in the case of PeEn, we set τ=1 for simplicity. Then, the Shannon Entropy (ShEn) is applied to the probabilities of the dispersion patterns in a similar way to the implementation of PeEn where permutations are used:

(9) $$DiEn=-\sum_{j}^{c^{m}}p\left(\pi_{j}\right)log\left(p\left(\pi_{j}\right)\right)$$

It is important to note that the number of possible dispersion patterns that can be assigned to each time series is set to $c^{m}$ as this links two main hyperparameters in the application of DiEn and creates a constraint on such values because $c^{m}$ < *N* (where *N* is the number of CAN-bus messages payload bytes in a sample in our specific problem). This is further discussed in Section 3.13.

For reproducibility of the results presented in this paper, the MATLAB implementation of the Dispersion Entropy provided by the authors in [19,29] was used in this paper.

### 3.10. Approximate Entropy

ApEn was initially proposed by Pincus in [30] and it is related to the predictability or regularity of a time series. It was devised as an approximation of the Kolmogorov entropy of an underlying process.

The algorithm to define ApEn is a search for the repetitive patterns of length *m* commencing at sample *i* in which the distance induced by the maximum norm differs up to an error threshold *r*.

Then, ApEn is defined by the following equation:

(10) $$ApEn\left(m,r,N\right)=\Phi^{m}\left(r\right)-\Phi^{m+1}\left(r\right)$$

where:

(11) $$\Phi^{m}\left(r\right)=\frac{1}{\left(N-m+1\right)}\sum_{i=1}^{N-m+1}log\left(C_{i}^{m}\left(r\right)\right)$$

with ${C_{i}}^{m}\left(r\right)$ is the number of vectors $x_{i}\in {ℜ}^{m}$ such that the distance $d(x_{i},x_{j})<r$ and $x_{i}=x_{i},x_{i+1},\ldots ,x_{i+m-1}$;

For reproducibility of the results presented in this paper, the MATLAB implementation of the Approximate Entropy provided by the authors in [31,32] was used in this paper.

### 3.11. Sample Entropy

SaEn was defined as an evolution of ApEn with the objective to solve the bias of ApEn due to counting self-matches and it was shown to exhibit better statistical properties than ApEn in many cases [33]. It is computed in a similar fashion than ApEn described in the previous Section 3.10, but the final step of calculating SaEn becomes:

(12) $$SaEn\left(m,r,N\right)=-log\left(\frac{A^{m}\left(r\right)}{B^{m}\left(r\right)}\right)$$

where $B_{m}\left(r\right)$ is defined as the mean of the number of vectors $x_{i}\in {ℜ}^{m}$ such that the distance $d(x_{i},x_{j})<r$ with i≠j divided by (N−m+1). The value of $A_{m}\left(r\right)$ is defined in the same way with $x_{i}\in {ℜ}^{m+1}$.

For the reproducibility of the results presented in this paper, the MATLAB implementation of the Sample Entropy provided by the authors in [33,34] was used in this paper.

### 3.12. Fuzzy Entropy

This paper also applies the Fuzzy Entropy (FzEn) defined by the authors in [35], where is reported that even if SaEn is slightly faster than FzEn, the latter is more consistent and less dependent on the data length of the series where it is applied. Fuzzy Entropy is based on similar concept of SaEn and ApEn but the number of vectors which satisfy the distance condition in comparison to the tolerance *r* is calculated using a fuzzy function of this form:

(13) $$\mu =e^{{-d}^{p}/r}$$

where *p* is set to 1 in the analysis done in this paper.

Then the FzEn is calculated as:

(14) $$FzEn\left(m,p,r,N\right)=-log\left(\frac{\alpha^{m}\left(r\right)}{\beta^{m}\left(r\right)}\right)$$

where α is related to ${ℜ}^{m+1}$ and β is related to ${ℜ}^{m}$

For reproducibility of the results presented in this paper, the MATLAB implementation of the Fuzzy Entropy provided by the authors in [34] was used in this paper.

### 3.13. Choice of the Hyperparameters for the Entropy Measures

**Figure 5:**
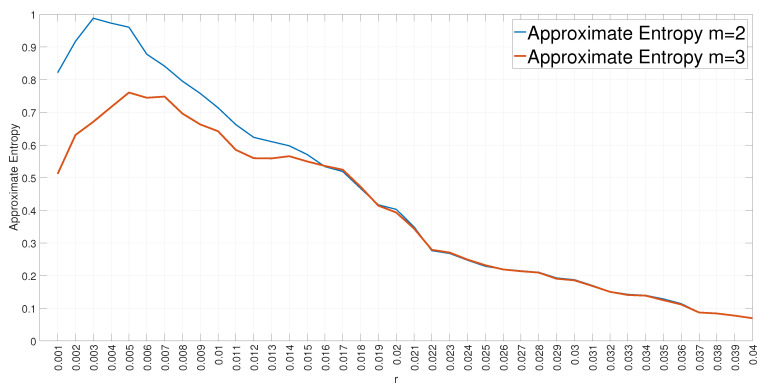
Approximate Entropy for m = 2 and m = 3 with varying r in the GEAR attack with $W_{s}=72$ and $R_{TT}=3/4$.

Each entropy measure identified above is based on the definition of hyperparameters. This section discuss the choice of the value of the hyperparameters in relation to the length N of the sequence of windowed data. In this specific proposal, the value of N in a sample is defined by $W_{s}$× 8 because all the CAN-bus messages in the data set have a full payload of 8 bytes. Apart from ShEn, all the other entropy measures are based on various hyperparameters: α for ReEn, the embedding dimension *m* for PeEn, DiEn, SaEn, ApEn and FzEn, the parameter *r* for SaEn, ApEn and FzEn and the parameter *c* for DiEn. In general, the studies presented in the literature [14,30,36] recommend that N>>m, to have a significant number of patterns to estimate the entropy. DiEn is also based on the parameter *c* or number of classes. In [19], the authors recommend that $N>c^{m}$. An increase on the value of m does also increase the computing time in the application of entropy features, which goes against the principle of this paper to make the detection process time-efficient. The values of m=2,3 are used as shown in the Results Section 4. Then, ApEn, SaEn and FzEn are also based on the tolerance or threshold value *r*. In literature [33], it is recommended to use values of $r>0.1*\sigma (X_{N})$ where $\sigma (X_{N})$ is the standard deviation of the series $X_{N}$ of payload bytes in a sample, but such value of r may change depending on the specific characteristics of $X_{N}$. Then, in this paper, the value of *r* is determined using the approach presented in [36] and a similar analysis has been conducted to evaluate when the maximum values of the Approximate Entropy are reached in relation to the ratio between the *r* and the standard deviation. One example on the application of the Approximate Entropy with window size $W_{s}=72$, $R_{TT}=3/4$ and the Gear attack is shown in Figure 5. Similar results are obtained for the other attacks and the other window sizes. Then, the values of r/σ in the range (0.01,..., 0.04) are used for the Approximate Entropy. A similar approach is used for SaEn and FzEn and a value of r/σ>0.1 is used. Finally, the values of α for ReEn are (1–4) as they are the same values adopted in [9] and it was experimentally found that values of α greater than 4 did not significantly improve detection.

Then, on the basis of the previous definitions of the entropy measures, we identify in Table 2 the list of the features used in the study with the related hyperparameters. As it can be seen by Table 2, a total of 34 features are defined and used to calculate the results presented in Section 4. This is a much larger set of entropy measures than what it is used in the literature for in-vehicle detection attacks.

## 4. Results

The aim of this section is to provide the results on the approach for the four different attacks (DoS, Gear, Fuzzy, RPM) present in the data set based on the different hyperparameters. Most of the analysis is conducted for the normal traffic estimate and the training phase with the objective to identify the optimal hyperparameters (i.e., $Fac_{thr}$, $RR_{TT}$ and feature id) over the entire data set. This objective is the focus of the first three subsections of this Section: Section 4.1–Section 4.3. In detail, Section 4.1 evaluates the classification performance of each entropy measure for all the four attacks. Section 4.2 provides the precision and recall for each entropy measure for a specific values of the threshold $Fac_{thr}$ by changing the values of the window size $W_{s}$ and the ratio $R_{TT}$. An example of the obtained values for False Positives and False Negatives is also provided. Section 4.3 analyzes the impact of $W_{s}$ and $R_{TT}$ on the classification performance of the Approximate Entropy, which is identified in the previous section as an optimal feature from the classification performance point of view. Then, the Section 4.4 provides the results for the detection phase, where the entire data set for all the four attacks is split in three portions for each phase on the basis of the ratio value $R_{TT}$: (1) the first portion, which is equal to the fraction $R_{TT}$ of the entire data set is used for Normal traffic estimate, (2) half of the (1-$R_{TT}$) portion of the entire data set is used for Training and (3) the remaining half of the (1-$R_{TT}$) portion of the entire data set is used for the Detection phase. Finally, the last subSection 4.5 describes the computing resources used in the analysis and provide the computing time for each of the three phases.

### 4.1. Evaluation of the Accuracy for Each Entropy Measure

**Figure 6:**
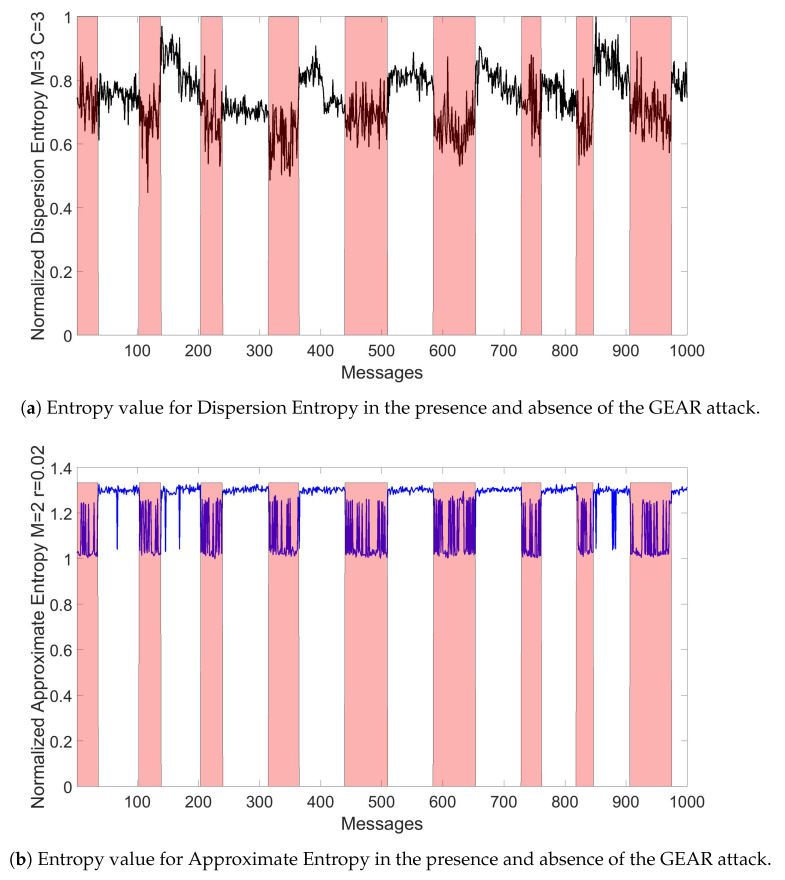
Entropy values for different entropy measures in the presence and absence of the GEAR attack with $W_{s}=72$ and $R_{TT}=3/4$.

In an initial step, it was evaluated how the entropy measures change when an attack is executed. It was found that some entropy measures provides more discriminating power in comparison to other entropy measures. In the proposed approach, this means that the range of values reported by the application of the specific entropy measure is significantly different in the legitimate traffic in comparison to the malicious traffic. An example is shown in Figure 6a,b respectively for the Dispersion Entropy and the Approximate Entropy in the presence of the Gear attack for $W_{s}=72$ and for $R_{TT}=3/4$. The figures only show a small segment of the overall in-vehicle traffic, which has been evaluated. The pink (or light gray in a b/w representation of this paper) bars represent the set of messages when the attack is implemented (i.e., malicious traffic), while the plot shows the calculated entropy measure. It can be seen that these two specific entropy measures have a significant discriminating power because the range of values is significantly different in the legitimate traffic from the malicious traffic: the mean of the entropy measure in the presence of legitimate traffic is quite different from the mean of the entropy measure in the presence of malicious traffic. Then, an high detection accuracy is possible even for relatively small values of $Fac_{thr}$.

**Figure 7:**
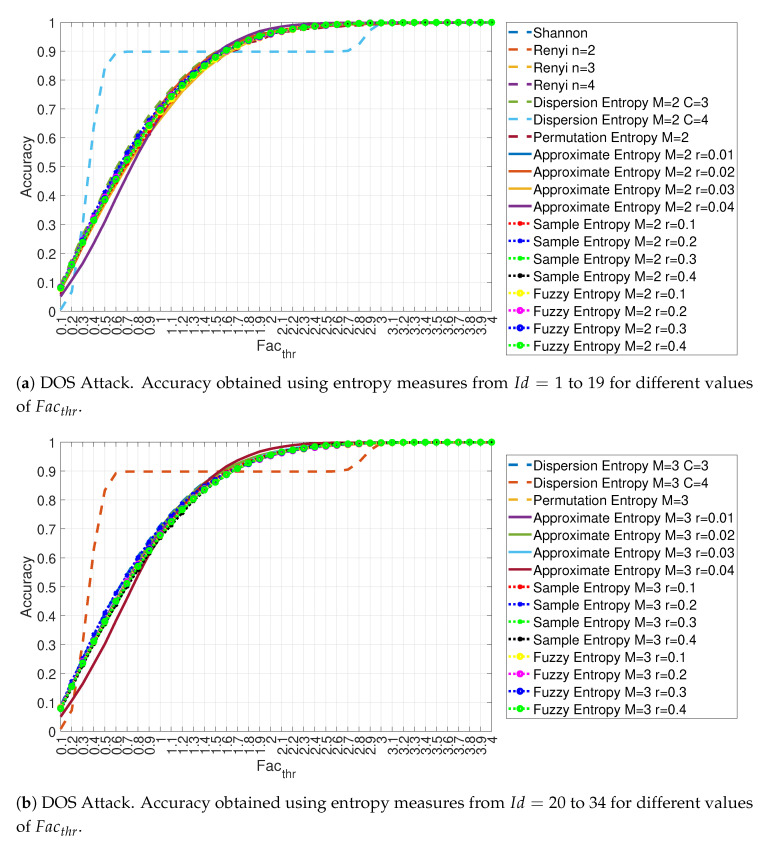
Accuracy obtained using different entropy measures for the DOS attack with $W_{s}=72$ and $R_{TT}=3/4$.

Not all the entropy measures provide the same clear visual distinction between legitimate and malicious traffic. Then, an extensive analysis of all the 34 entropy measures for the four attacks was performed. Figure 7a,b show the accuracy of the proposed approach at the variation of the parameter $Fac_{thr}$ between 0 and 4 in 0.1 steps of $\sigma_{i}$ for the DoS attack and $R_{TT}=3/4$. Because of the large number of features, two pictures were created: Figure 7a for entropy measures feature id from Id=1 to 19 and Figure 7b for entropy measures feature id from Id=20 to 34. The results are consistent with the literature where a low value of the threshold $Fac_{thr}$ leads to a limited detection performance. It can be seen that for $Fac_{thr}$ approaching 4, the detection accuracy is very high, and it eventually reaches almost 100% detection accuracy. This is also consistent with literature because DoS attacks impact significantly the entropy values calculated on the in-vehicle traffic. The figures shows also that most of the entropy measures exhibit a similar detection performance with the significant difference of the Dispersion Entropy with c = 4 both with m = 2 (Feature Id = 6 in Figure 7a) and m = 3 (Feature Id = 21 in Figure 7b). A potential explanation of this deviation is that with C = 4 the calculation of the Dispersion Entropy is noisier than with c = 3 thus leading to a divergent behavior.

**Figure 8:**
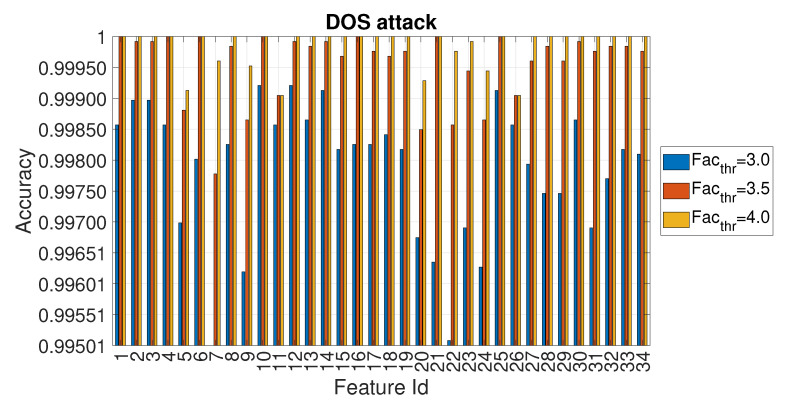
Accuracy in relation to the feature id for values $Fac_{thr}$ equal to 3.0, 3.5 and 4.0 for the DOS attack with $W_{s}=72$ and $R_{TT}$ = 3/4.

Because the accuracy values reported in Figure 7a,b are quite similar, the following Figure 8 provides a more detailed view of the accuracy obtained for each feature id (in this type of attack, the accuracy values are nevertheless quite near) for three selected values of $Fac_{thr}$.

**Figure 9:**
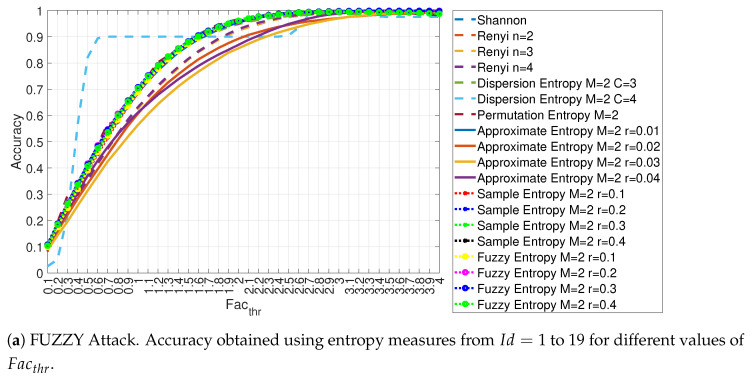

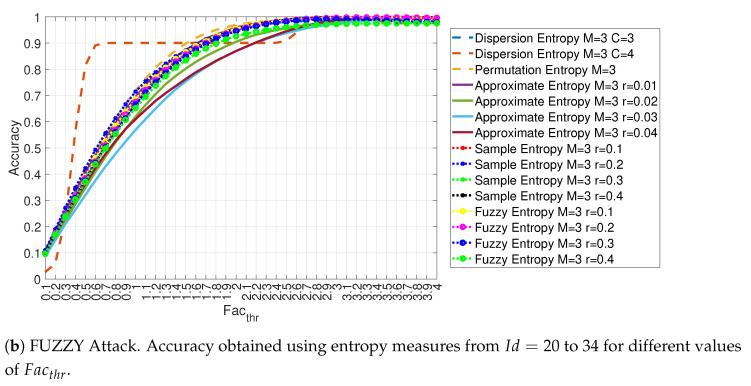
Accuracy obtained using different entropy measures for the FUZZY attack with $W_{s}=72$ and $R_{TT}$ = 3/4.

Similar results to the ones obtained for the DoS attack are also obtained with the Fuzzy attack as shown in Figure 9a,b respectively for the entropy measures from Id=1 to 19 and from Id=20 to 34. We see a similar behavior than the one obtained for the DoS attack where for high values of $Fac_{thr}$, it is possible to obtain a very high detection accuracy near 100% and most of entropy measures performs in a similar way at the increase of the value of $Fac_{thr}$. As with the DoS attack, the Dispersion Entropy with c = 4 has a different behavior from the other entropy measures, obtaining a high value of detection accuracy for relative (in comparison to the other entropy measures) limited values of $Fac_{thr}$ but eventually converging to the other entropy measures for very high values (i.e., $Fac_{thr}$ approaching 4). For high values of thresholds both DoS and Fuzzy attacks can be detected with almost 100% accuracy obtaining similar results to ones obtained on the same data set with more sophisticated techniques like Deep Learning [27]. The reason is that both attacks generate traffic, which is significantly different from the normal in-vehicle CAN-bus traffic and entropy measures are able to detect such anomalous behavior if the threshold is large enough.

**Figure 10:**
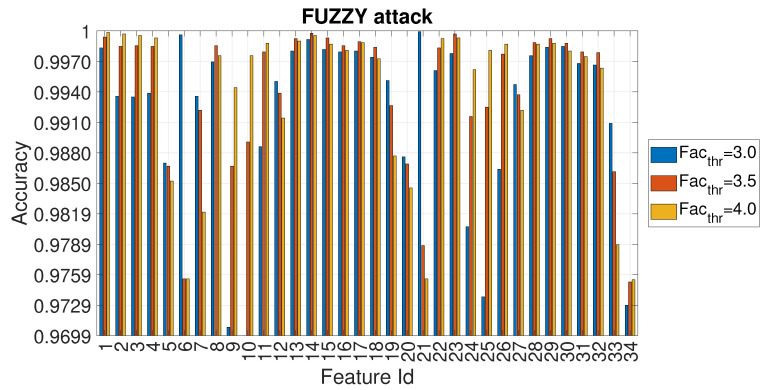
Accuracy in relation to the feature id for values $Fac_{thr}$ equal to 3.0, 3.5 and 4.0 for the FUZZY attack with $W_{s}=72$ and $R_{TT}$ = 3/4.

In a similar way to the DoS attack, the values of accuracy for each feature id are reported for three selected values of $Fac_{thr}$ in Figure 10 for the Fuzzy attack.

As shown in the following results, the detection of spoofing attacks is more challenging because the injected malicious messages are quite similar to legitimate operations (e.g., related to the functioning of the gear).

**Figure 11:**
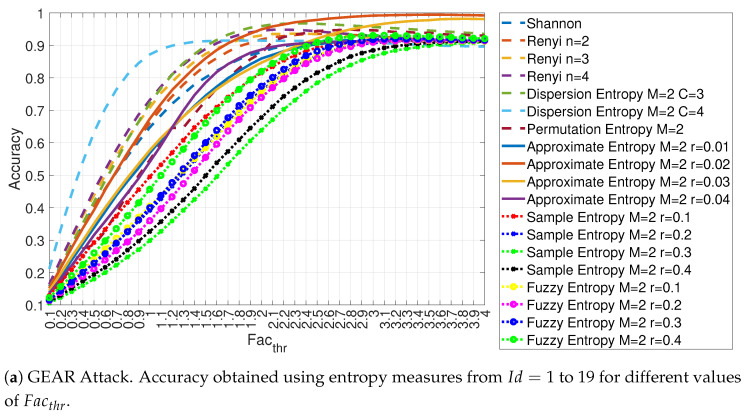

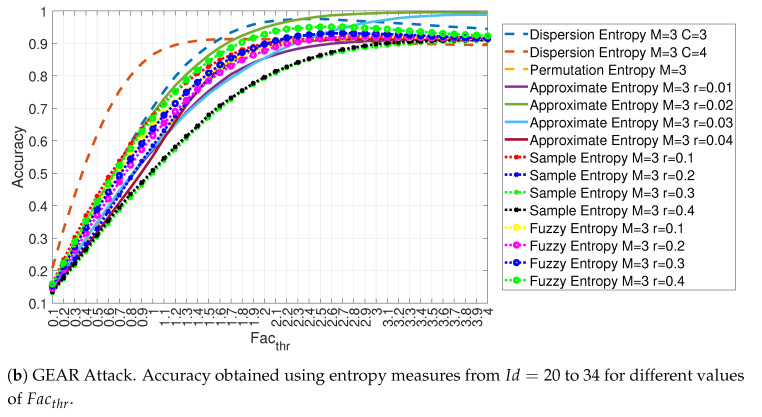
Accuracy obtained using different entropy measures for the GEAR attack with $W_{s}=72$ and $R_{TT}$ = 3/4.

This assumption can be validated by the results presented in Figure 11a,b for the Gear attack. One initial observation is that increasing the threshold value $Fac_{thr}$ to the limit of 4 does not always lead to the optimal detection accuracy for all the entropy measure as some entropy measures presents an optimal values well below $Fac_{thr}=4$. Then, these results are significant, because they show that the value of $Fac_{thr}$ must be chosen in an appropriate way. The second and more important observation is that the detection performance of each entropy measure is significantly different from each other. In particular, Approximate Entropy with values of m = 2 and r = 0.02 and r = 0.03 (respectively Id = 9 and Id = 10) and Approximate Entropy with values of m = 3 and r = 0.02 and r = 0.03 (respectively Id = 24 and Id = 25) are able to reach almost 100% detection accuracy for $Fac_{thr}=4$ (which is also their optimal value) while the proposed approach based on specific entropy measures is not able to reach an high detection accuracy. The Dispersion Entropy measures are able to reach an higher classification accuracy than the other entropy measures for low values of $Fac_{thr}$, but then they reach a plateau around 90% detection accuracy even if the values of $Fac_{thr}$ is increased to the maximum value of 4. The classification based on Sample Entropy provides the worst results among all for this specific type of attack in particular for values of m = 2. The Shannon Entropy and Renyi Entropy used in the literature [9] are in the middle of a ranking of the entropy measures and they exhibit an optimal detection accuracy for a value of the threshold $Fac_{thr}$ slightly above 2. It is noted that the Dispersion Entropy has the best accuracy for relatively low values of $Fac_{thr}$, but then it reaches a peak and the accuracy decreases for increasing values of $Fac_{thr}$ as many other features. The reason for the behavior that the accuracy reaches a maximum and then decrease of higher values of $Fac_{thr}$ is that an increase of the value of $Fac_{thr}$ forces the algorithm to include samples containing CAN-bus messages of the RPM and Gear attacks. Because $Fac_{thr}$ is related to the standard deviation of the legitimate in-vehicle CAN-bus traffic, this behavior can be explained by looking again at the example of entropy measures of Dispersion Entropy and Permutation Entropy shown in Figure 6. In particular, Figure 6a shows the large variation of the values of Dispersion Entropy in normal traffic. Then, larger values of $Fac_{thr}$ may include sample related to attacks causing the algorithm to lose accuracy as the number of FP may increase. This may explain why the accuracy plot in Figure 11 reaches a maximum and then slowly degrades. On the other side, Figure 6b for the Approximate Entropy shows that the values of the calculated entropy are in tight range (i.e., small values of standard deviation). Then, even when $Fac_{thr}$ is approaching the value of 4, the algorithm can discriminate with high accuracy legitimate samples from samples containing malicious CAN-bus messages.

**Figure 12:**
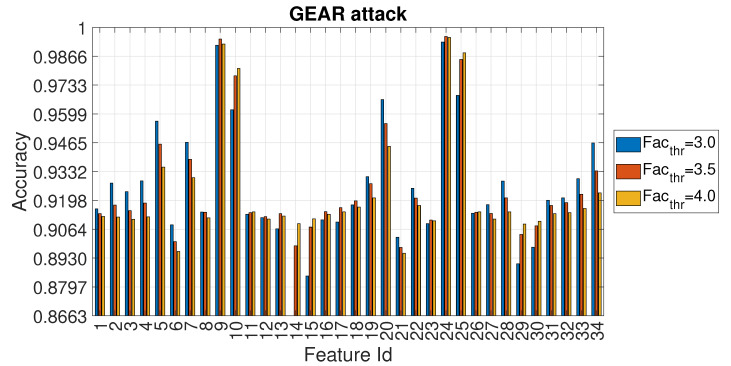
Accuracy in relation to the feature id for values $Fac_{thr}$ equal to 3.0, 3.5 and 4.0 for the GEAR attack with $W_{s}=72$ and $R_{TT}$ = 3/4.

The detailed values of the accuracy for $Fac_{thr}$ equal to 3.0, 3.5 and 4.0 are shown for each feature in Figure 12.

**Figure 13:**
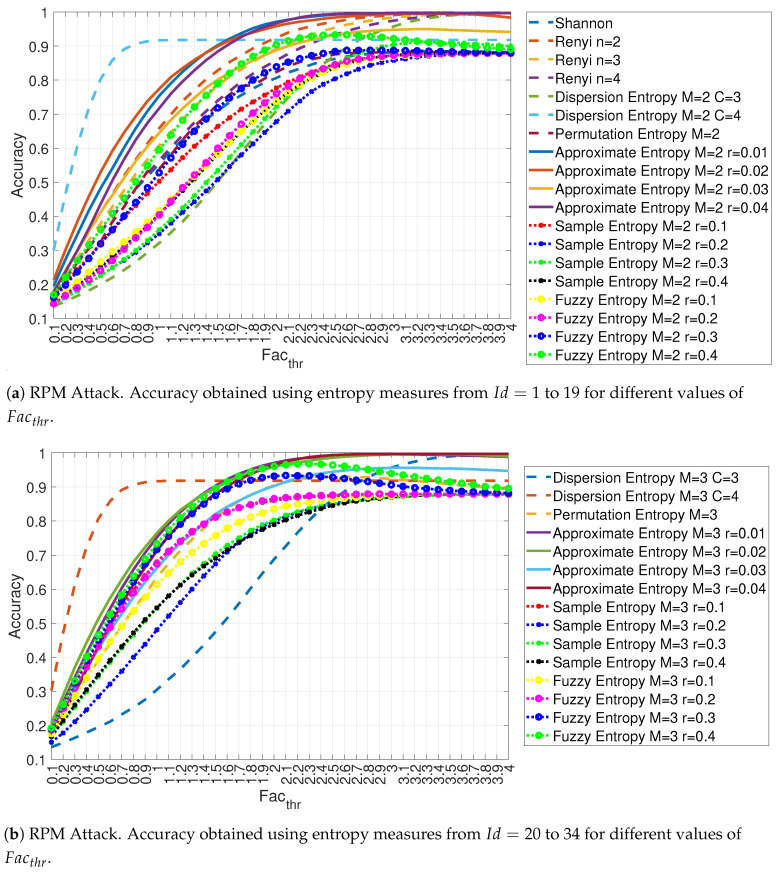
Accuracy obtained using different entropy measures for the RPM attack with $W_{s}=72$ and $R_{TT}$ = 3/4.

Similar results are obtained for the other spoofing attack: the RPM attack as shown in Figure 13a,b. The choice of the entropy measure affects significantly the accuracy performance, with the Fuzzy Entropy measures performing worse than the other entropy measures and the Approximate Entropy providing the best accuracy.

To summarize, the Gear and RPM attacks are more difficult to identify in comparison to the DoS and Fuzzy attack. In addition, Gear and RPM attacks require tuning and careful choice of the entropy measure and the optimization values of $Fac_{thr}$ because some entropy measures are never able to reach very high accuracy (e.g., 99%) even for high thresholds and not necessarily the highest value of $Fac_{thr}$ is able to provide the optimal detection accuracy. To highlight more these significant results, Table 3 presents the optimal values for each entropy measure and the corresponding value of $Fac_{thr}$ where the optimal accuracy value is obtained. We note that these results were obtained with $W_{s}=72$ and $R_{TT}=3/4$. Similar results were obtained with the other values of $W_{s}$ and $R_{TT}$ but they are not provided here for lack of space. The impact of $W_{s}$ and $R_{TT}$ is investigated in the next subsections.

**Figure 14:**
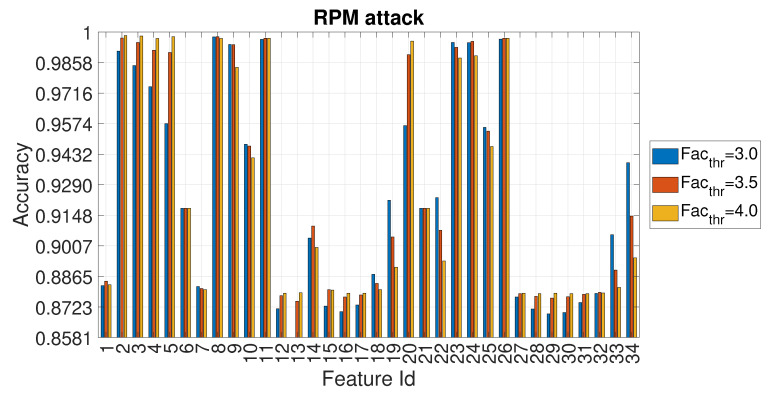
Accuracy in relation to the feature id for values $Fac_{thr}$ equal to 3.0, 3.5 and 4.0 for the RPM attack with $W_{s}=72$ and $R_{TT}$ = 3/4.

As for the previous results, Figure 14 shows the detailed values of the accuracy for $Fac_{thr}$ equal to 3.0, 3.5 and 4.0 for the RPM attack.

### 4.2. Recall and Precision for the Gear Attack

The aim of this subsection is to analyze how the recall and precision changes at the variation of $W_{s}$ and $R_{TT}$ for each of the entropy measures for the specific value of the threshold $Fac_{thr}=2$.

**Figure 15:**
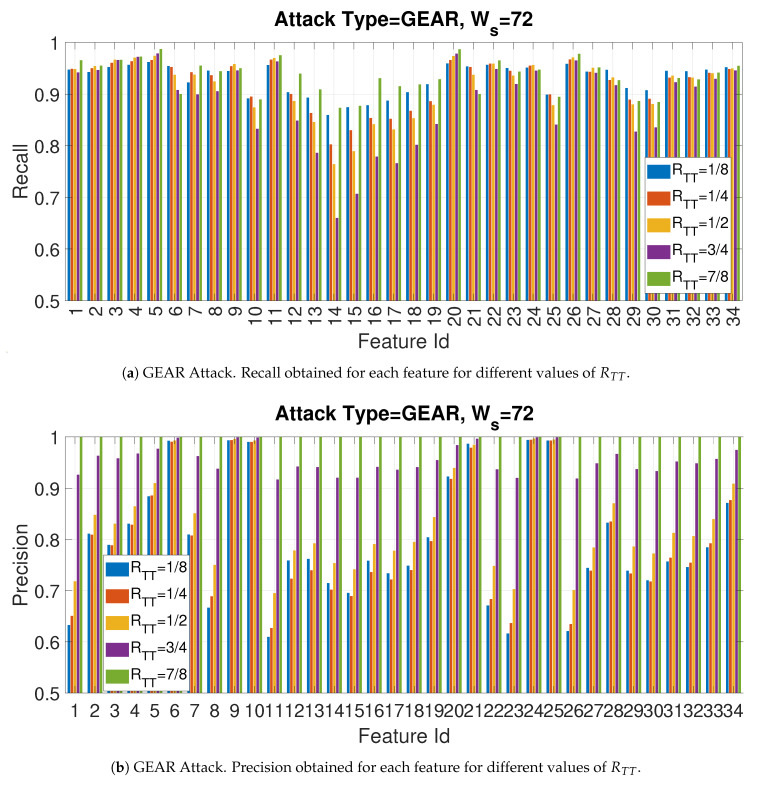
GEAR Attack. Precision and recall for different values of the ratio $R_{TT}$ with $Fac_{thr}\;=$ 2.

The Bar Figure 15a,b provide respectively the recall and precision for the Gear attack for each of the entropy measures for $Fac_{thr}=2$ and $W_{s}=72$ at the variation of the parameter $R_{TT}$. Please refer to Table 2 for the description of each entropy measure associated with the specific Id appearing on the X axis of Figures. Both Figures show different bars for each value of ratio $R_{TT}$. The result confirms the previous accuracy Figures (e.g., Figure 11), which shows that precision and recall can vary greatly among the entropy measures and the specific entropy measure must be carefully selected. The Figure 15a,b show that the balance between the size of the training set and the test set impacts both metrics but in particular the precision. It can be seen from Figure 15b that a larger training set (e.g., increasing value of $R_{TT}$) provides higher values in a consistent way across all the entropy measures. This result indicates an important design decision as a larger value of $RR_{TT}$ may provide more stable values of $u_{i}$ and $\sigma_{i}$ to support a more stable choice of the hyperparameters and an improved detection accuracy. In particular, the precision (as indicated before) is probably more relevant than the recall in this particular detection problem, as the goal is to minimize the FP where an intrusion is wrongly detected an legitimate traffic thus allowing the attacker to implement the cybersecurity threat. On the other side, Figure 15a shows that such trend is not the same across all the entropy measures. For example, the accuracy obtained with Feature Id = 10 (ApEn, m = 2 and r/σ=0.03) does not change significantly. In addition, as shown more in detail in Section 4.3, the improvement in classification performance due to the $R_{TT}$ depends both on the entropy measure but also the value of the threshold $Fac_{thr}$. Then, all these factors should be taken in consideration.

**Figure 16:**
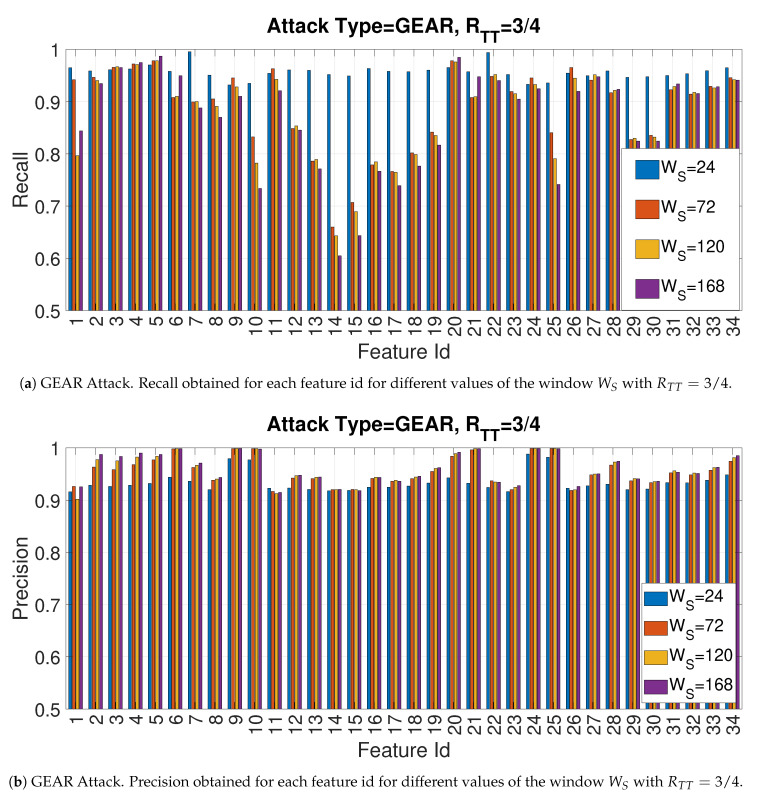
GEAR Attack. Precision and recall for different values of the window $W_{S}$ with $R_{TT}=3/4$ with $Fac_{thr}\;=$ 2.

In another phase of the study presented in this paper, the impact of the window size was evaluated. As in the previous case, only one specific attack is presented for space reasons. The Bar Figure 16a,b provide respectively the recall and precision for the Gear attack for each of the entropy measures for $Fac_{thr}=2$ and $R_{TT}=3/4$ and by changing the size of the window $W_{s}$. The size of window size is another important hyperparameter: a small sample size may require more time for training as the data set is segmented in a greater number of segments on which the entropy measure must be calculated (thus requiring more time), but it may provide higher detection accuracy because the CAN-bus messages related to a cybersecurity attack would have in percentage more weight in the sample. The latter aspect is confirmed by the Figure 16a,b because the recall is significantly higher for $W_{s}=24$ rather than the larger values of $W_{s}$. On the other side, the precision is slightly better with larger values of $W_{s}$.

**Figure 17:**
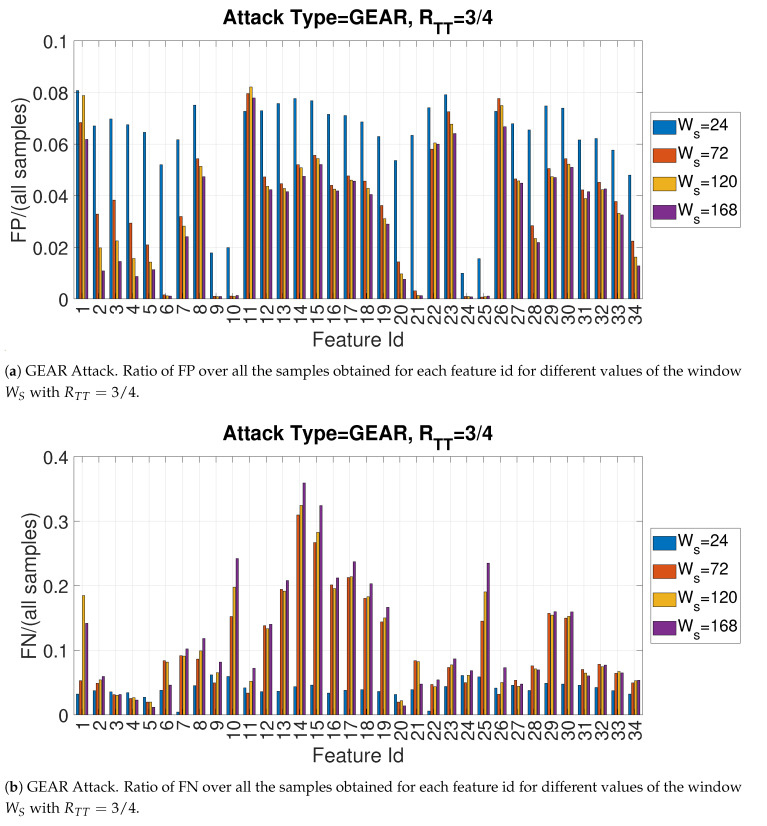
GEAR Attack. Ratios of FP and FN over all the samples for different values of the window $W_{S}$ with $R_{TT}=3/4$ and $Fac_{thr}\;=$ 2.

To complement the previous Figure 16a,b and to provide an independent evaluation of FN and FP, the following Figure 17a,b provide respectively the number of False Positives (FP) and False Negatives (FN) over all the samples.

### 4.3. Evaluation of Accuracy in Relation to $R_{Tt}$ and $W_{s}$ at the Variation of ${Fac}_{Thr}$

This subsection shows the impact of the value of the threshold $Fac_{thr}$ both for $R_{TT}$ and $W_{s}$. Two specific entropy measures (Id = 5 and Id = 10) are selected in relation to the specific GEAR attack.

**Figure 18:**
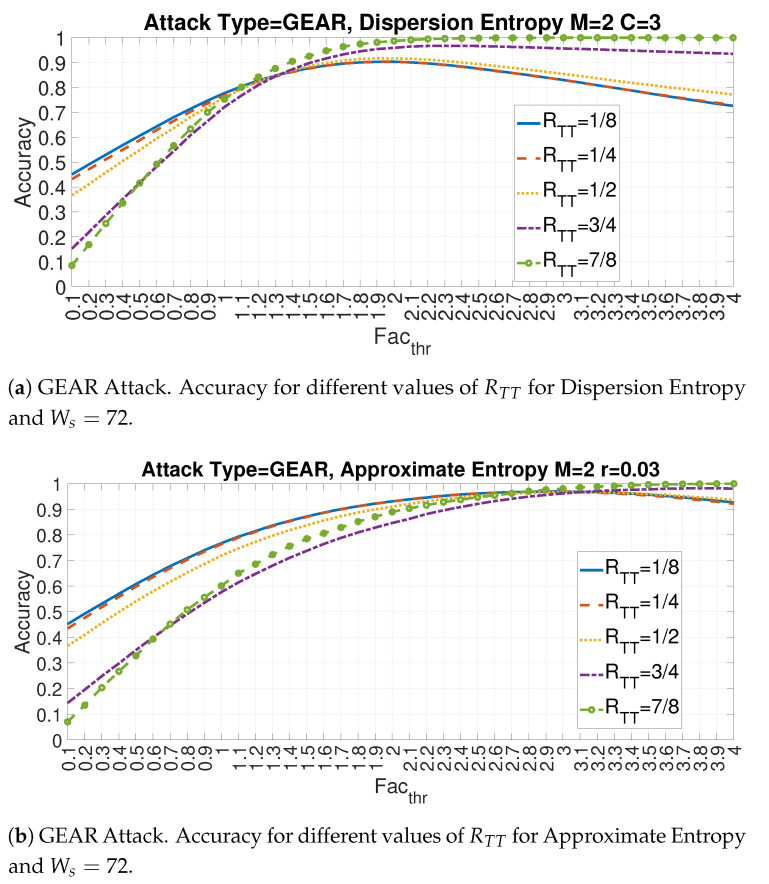
Impact of $Fac_{thr}$ on Accuracy for features Id = 5 and 10 for different values of $R_{TT}$ and $W_{s}=72$ for the GEAR attack.

The following Figure 18a,b provide the plots respectively for the Feature Id = 5 (Dispersion Entropy) and Feature Id = 10 (Approximate Entropy) for different values of the ratio $R_{TT}$ and $W_{s}$ = 72. Two main observations can be derived from Figure 18a,b. The first one is that the optimal value of $Fac_{thr}$ changes considerably with the value of $R_{TT}$ for both entropy measures (similar results are obtained for the other entropy measures but they are not displayed here for lack of space). Then, the combination of $R_{TT}$ and $Fac_{thr}$ must be carefully identified. The second observation confirms the previous results that the optimal detection accuracy is obtained with high values of $R_{TT}$. The larger is the portion of the data set is used to calculate mean and standard deviation and more accurate is the detection.

**Figure 19:**
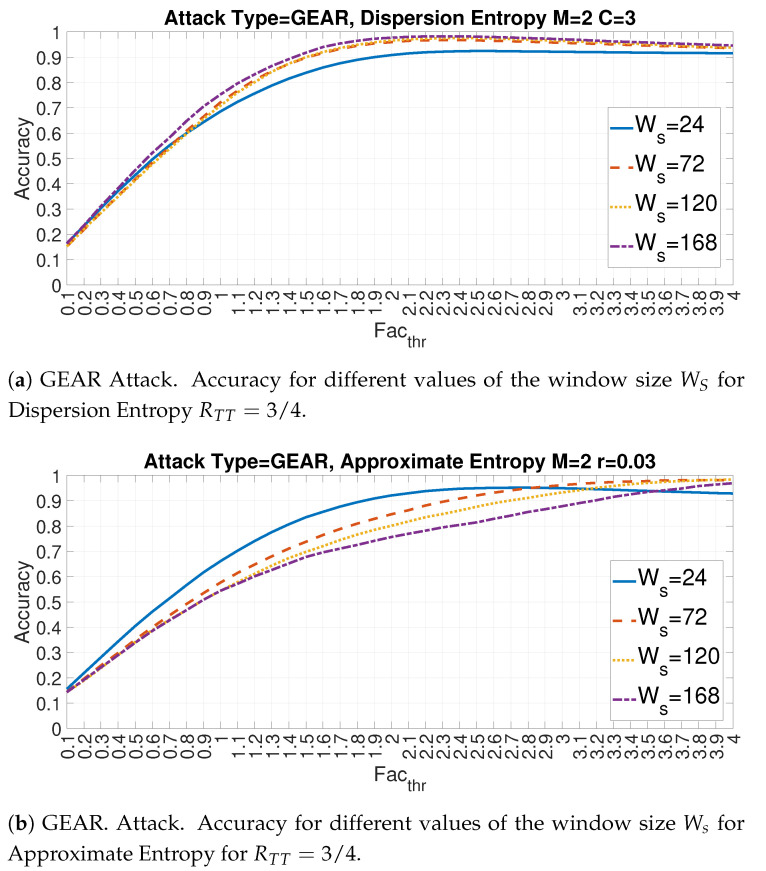
Impact of $Fac_{thr}$ on Accuracy for features Id = 5 and 10 for different values of $W_{S}$ and $R_{TT}=72$ for the GEAR attack.

The following Figure 19a,b provide the graphs respectively for the feature Id = 5 (Dispersion Entropy) and feature Id = 10 (Approximate Entropy) for different values of the window size $W_{S}$ and $R_{TT}=3/4$. In this case, the results shows that the impact of the $W_{s}$ can be different across entropy measures and the optimal values are obtained through a proper combination of $W_{s}$ with the entropy measure. In fact, in Figure 19a a smaller window size $W_{s}=24$ provides less detection accuracy than larger windows (e.g., $W_{s}=168$) for all the values of $Fac_{thr}$. For Figure 19b, a small window size of $W_{s}=24$ is able to provide the best accuracy for most of the values of $Fac_{thr}$ apart from values near 4, where larger windows sizes are more effective. A potential explanation for this behavior is related to the characteristics of each entropy measure. In particular Dispersion Entropy requires longer time series related to the $C^{m}$ condition to provide correct results while Approximate Entropy can correctly estimate entropy with shorter time series.

### 4.4. Detection

This section provides the results for the detection phase. Although the previous sections on the training was conducted on the entire data set, the evaluation of the detection phase is performed by splitting in half the remaining of the data set ($1-R_{TT}$ of the entire data set), which is not used for the normal traffic estimate ($R_{TT}$ of the data set). For example, if a value of $R_{TT}=1/2$ is used, the first half of the data set is used for the normal traffic estimate, one quarter is used for training and one quarter is used for detection. The calculation has been performed for all the different attacks (i.e., DoS, Fuzzy, Gear, RPM), for all the different sizes ($W_{s}=24,72,120,168$) and for different values of $R_{TT}$.

**Figure 20:**
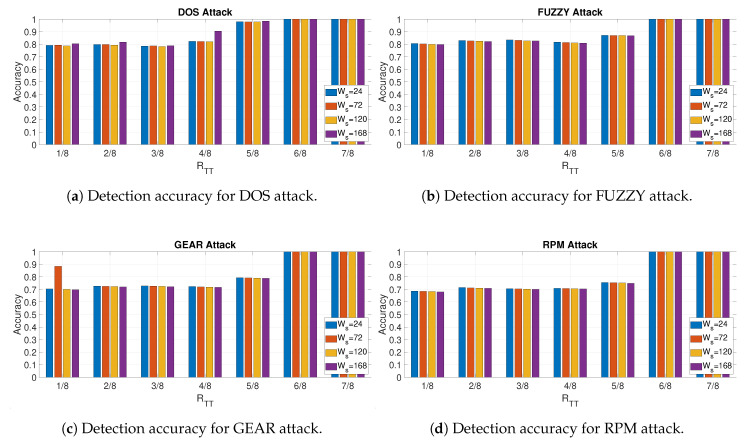
Detection accuracy for all different attacks and different values of $R_{TT}$ and $W_{S}$.

The results of the analysis are provided in Figure 20, while the values of the reported accuracy for all the attacks and $W_{s}=72$ are shown in Table 4 together with the optimal feature id and the optimal $Fac_{thr}$ from the Training Phase. In particular, Figure 20a–d show the accuracy respectively for the DoS, Fuzzy, Gear and RPM attacks at the variation of $R_{TT}$. The results are consistent with the results presented in the previous subsections of this section where lower values of $R_{TT}$ can provide a relatively low accuracy for the detection of the in-vehicle attack. When the amount of data used for the normal traffic estimate is larger (e.g., values of $R_{TT}$ higher than 0.5) the accuracy increases significantly. This trend is similar for all the attacks. It is noted that the accuracy has a sharp increase in particular for the Gear and RPM attacks (Figure 20c,d), which are more difficult to detect. Although this is consistent with the other results, it can also be based on the consideration that for such high values of $R_{TT}$, the driving circumstances were very similar for the training and the detection phases; then it is easier for the algorithm to detect attacks because the optimal features and thresholds used for the detection were calculated in similar driving circumstances, thus explaining the very high accuracy. When the training and detection phase are based on the analysis of a relative large set of data (lower values of $R_{TT}$), the driving circumstances may be different thus lowering the detection accuracy. Future developments of the research presented in this paper, could evaluate methods of statistical analysis, which take in consideration and identify different optimal features and thresholds for different driving circumstances. Such analysis could be quite complex because it must take in consideration the range of different driving circumstances, identify in which driving circumstances the detection is currently executed and it must choose the appropriate optimal features and thresholds. This complex analysis is out of the scope of this paper and it is reserved for future developments (see Conclusions Section 5).

Table 4 provides additional information to the Figure 20 as it identifies the optimal feature ids and values of $Fac_{thr}$ from the training phases, which are used for the detection phase. The results are consistent with the previous sections where Approximate Entropy (feature Id = 24) and Dispersion Entropy (feature Id = 21) provides optimal results. Shannon Entropy is also the optimal feature id for the DoS and Fuzzy attacks. The optimal values of $Fac_{thr}$ are generally high (more than 2.9), which is also consistent with the previous results show in Section 4.1.

### 4.5. Computing Resources Used to Perform the Study

This section describes the computing resources used to perform the analysis and the time needed for each of the three phases for three specific ratios of the used data set. The computing platform used in the study is a mass market laptop with processor unit i7 8550U CPU 1.8 GHz with 8 Gigabytes of RAM. Table 5 shows the computing time for selected attacks and parameters ($R_{TT}=1/4$) ($W_{s}=72$). The provided times in Table 5 are based on the processing of the entropy features already calculated on the CAN-bus messages. The calculation of the entropy measures is estimated to be 40 s for 10,000 CAN-bus messages for the Normal Traffic estimate and Initial Training phase, while it is in range 0.45 s to 1.6 s for 10,000 CAN-bus messages in the Detection phase because a single entropy measure (the optimal entropy measure calculated from the Training phase) has to be calculated in this phase. From these values and the values reported in Table 5, it can be concluded that the Detection phase can be quite fast even using a mass market processor unit, while the Training phase can take considerable time because of the need to calculate the performance of all the potential entropy measures across a wide range of thresholds. From a practical deployment point of view, the Normal Traffic estimate and Training phase could be performed by a powerful cloud computing facility, while the detection phase must be performed in the vehicle itself.

## 5. Conclusions

This paper has evaluated the application of different entropy measures using a sliding window to the analysis of the payload of CAN-bus messages to identify in-vehicle attacks. Even if this approach has already been proposed in the literature where it has shown to be very time-efficient in comparison to other approaches, literature results have mostly focused on specific attacks or specific entropy measures. The analysis presented in this paper is based on an extensive range of entropy measures and different values of hyperparameters: window size, threshold range and the parameters, which are part of the definition of the entropy measures themselves (e.g., embedding dimension). The results show than an adequate selection of the entropy measures and the value of the hyperparameters can provide a very high detection accuracy. The results are based on a public data set with millions of records and four different attacks and they can be used to support further research in this area.

Future developments will investigate a more complex analysis, which takes in consideration the specific driving circumstances and identify different sets of the parameters (mean, standard deviation, feature and thresholds) for each driving circumstance, which are then applied to the detection phase.

## Figures and Tables

**Table 1:** Data set used in this paper from [26,27].

Attack Type	Number of Messages	Number of Normal Messages	Number of Injected Messages
DoS Attack	3,665,771	3,078,250	587,521
Fuzzy Attack	3,838,860	3,347,013	491,847
Spoofing the drive Gear	4,443,142	3,845,890	597,252
Spoofing the RPM gauge	4,621,702	3,966,805	654,897

**Table 2:** List of features used in the analysis (σ is the standard deviation of the sample).

Feature Id	Description of the Feature	Hyperparameter
1	Shannon Entropy	None
2	Renyi Entropy	α=2
3	Renyi Entropy	α=3
4	Renyi Entropy	α=4
5	Dispersion Entropy	m = 2, c = 3
6	Dispersion Entropy	m = 2, c = 4
7	Permutation Entropy	m = 2
8	Approximate Entropy	m = 2, *r* = 0.01 × σ
9	Approximate Entropy	m = 2, *r* = 0.02 × σ
10	Approximate Entropy	m = 2, *r* = 0.03 × σ
11	Approximate Entropy	m = 2, *r* = 0.04 × σ
12	Sample Entropy	m = 2, *r* = 0.1 × σ
13	Sample Entropy	m = 2, *r* = 0.2 × σ
14	Sample Entropy	m = 2, *r* = 0.3 × σ
15	Sample Entropy	m = 2, *r* = 0.4 × σ
16	Fuzzy Entropy	m = 2, *r* = 0.1 × σ
17	Fuzzy Entropy	m = 2, *r* = 0.2 × σ
18	Fuzzy Entropy	m = 2, *r* = 0.3 × σ
19	Fuzzy Entropy	m = 2, *r* = 0.4 × σ
20	Dispersion Entropy	m = 3, c = 3
21	Dispersion Entropy	m = 3, c = 4
22	Permutation Entropy	m = 3
23	Approximate Entropy	m = 3, *r* = 0.01 × σ
24	Approximate Entropy	m = 3, *r* = 0.02 × σ
25	Approximate Entropy	m = 3, *r* = 0.03 × σ
26	Approximate Entropy	m = 3, *r* = 0.04 × σ
27	Sample Entropy	m = 3, *r* = 0.1 × σ
28	Sample Entropy	m = 3, *r* = 0.2 × σ
29	Sample Entropy	m = 3, *r* = 0.3 × σ
30	Sample Entropy	m = 3, *r* = 0.4 × σ
31	Fuzzy Entropy	m = 3, *r* = 0.1 × σ
32	Fuzzy Entropy	m = 3, *r* = 0.2 × σ
33	Fuzzy Entropy	m = 3, *r* = 0.3 × σ
34	Fuzzy Entropy	m = 3, *r* = 0.4 × σ

**Table 3:** Optimal Accuracy results for GEAR and RPM attacks in relation to $Fac_{thr}$ with $W_{s}=72$.

Feature Id	GEAR Accuracy	GEAR ${\mathrm{Fac}}_{\mathrm{thr}}$	RPM Accuracy	RPM ${\mathrm{Fac}}_{\mathrm{thr}}$
1	0.916	2.9	0.885	3.4
2	0.935	2.5	0.998	3.9
3	0.936	2.4	0.997	4.0
4	0.948	2.2	0.918	4.0
5	0.967	2.4	0.997	4.0
6	0.915	1.8	0.918	1.3
7	0.948	2.8	0.882	3.2
8	0.915	3.5	0.998	3.4
9	0.994	3.8	0.995	3.3
10	0.981	3.8	0.949	3.2
11	0.914	3.2	0.997	3.5
12	0.913	3.2	0.878	4.0
13	0.913	3.4	0.879	4.0
14	0.909	4.0	0.912	3.3
15	0.911	3.9	0.881	3.7
16	0.915	3.6	0.878	3.9
17	0.916	3.4	0.878	3.8
18	0.920	3.2	0.887	2.9
19	0.931	3.1	0.933	2.5
20	0.975	2.4	0.995	4.0
21	0.913	1.8	0.918	1.2
22	0.928	2.6	0.930	2.6
23	0.910	3.4	0.995	3.1
24	0.996	3.7	0.996	3.3
25	0.988	3.9	0.956	3.1
26	0.914	3.8	0.997	3.5
27	0.919	2.9	0.878	3.7
28	0.932	2.7	0.878	4.0
29	0.908	4.0	0.878	4.0
30	0.910	3.9	0.878	4.0
31	0.920	2.9	0.878	3.8
32	0.922	2.9	0.879	2.9
33	0.931	2.7	0.933	2.1
34	0.951	2.5	0.967	2.3

**Table 4:** Accuracy obtained in the Detection phase for different attacks and different values of $R_{TT}$.

Attack (Ratio), (Window size)	Accuracy	Precision	Recall	Optimal Feature ID from Training Phase	Optimal ${\mathrm{Fac}}_{\mathrm{thr}}$ from Training Phase
DOS ($R_{TT}=1/4$), ($W_{s}=72$)	0.817	0.812	0.995	1	3.9
DOS ($R_{TT}=2/4$), ($W_{s}=72$)	0.904	0.895	0.995	1	3.5
DOS ($R_{TT}=3/4$), ($W_{s}=72$)	0.995	0.996	0.998	34	3.9
FUZZY ($R_{TT}=1/4$), ($W_{s}=72$)	0.822	0.822	0.996	21	3.9
FUZZY ($R_{TT}=2/4$), ($W_{s}=72$)	0.809	0.809	0.994	1	4.0
FUZZY ($R_{TT}=3/4$), ($W_{s}=72$)	0.992	0.996	0.994	1	3.6
GEAR ($R_{TT}=1/4$), ($W_{s}=72$)	0.719	0.719	0.995	24	2.9
GEAR ($R_{TT}=2/4$), ($W_{s}=72$)	0.716	0.716	0.996	24	3.5
GEAR ($R_{TT}=3/4$), ($W_{s}=72$)	0.996	0.998	0.997	24	3.7
RPM ($R_{TT}=1/4$), ($W_{s}=72$)	0.709	0.708	0.993	4	3.5
RPM ($R_{TT}=2/4$), ($W_{s}=72$)	0.703	0.703	0.997	9	3.2
RPM ($R_{TT}=3/4$), ($W_{s}=72$)	0.991	0.998	0.997	24	3.7

**Table 5:** Processing time for GEAR and RPM attacks for different phases in seconds (s).

Attack (Ratio) and (Window Size)	Normal Traffic Estimate	Initial Training Phase	Detection Phase
GEAR ($R_{TT}=1/4$) ($W_{s}=72$)	0.0414 s	94.95 s	0.0523 s
RPM ($R_{TT}=1/4$) ($W_{s}=72$)	0.04 s	230.11 s	0.08 s
GEAR ($R_{TT}=3/4$) ($W_{s}=72$)	0.0496 s	22.06 s	0.0024 s
RPM ($R_{TT}=3/4$) ($W_{s}=72$)	0.0628 s	32.78 s	0.002 s

## Abbreviations

The following abbreviations are used in this manuscript:

CAN	Controller Area Network
CAN-bus	Controller Area Network-bus
ECU	Electronic Control Unit
DiEn	Dispersion Entropy
IDS	Intrusion Detection System
FP	False Positives
FN	False Negatives
ICT	Information and Communication Technologies
IDS	Intrusion Detection System
LiDAR	Light Detection and Ranging
NCA	Neighborhood Component Analysis
NCDF	Normal Cumulative Distribution Function
PeEn	Permutation Entropy
RBF	Radial Basis Function
SaEn	Sample Entropy
ShEn	Shannon Entropy
SVM	Support Vector Machine
TN	True Negatives
TP	True Positives

## References

[1] Checkoway S., McCoy D., Kantor B., Anderson D., Shacham H., Savage S., Koscher K., Czeskis A., Roesner F., Kohno T. Comprehensive experimental analyses of automotive attack surfaces. Proceedings of the USENIX Security Symposium.

[2] Petit J., Shladover S.E. (2014). Potential cyberattacks on automated vehicles. IEEE Trans. Intell. Transp. Syst..

[3] Marchetti M., Stabili D., Guido A., Colajanni M. Evaluation of anomaly detection for in-vehicle networks through information-theoretic algorithms. Proceedings of the 2016 IEEE 2nd International Forum on Research and Technologies for Society and Industry Leveraging a better tomorrow (RTSI).

[4] Al-Jarrah O.Y., Maple C., Dianati M., Oxtoby D., Mouzakitis A. (2019). Intrusion detection systems for intra-vehicle networks: A review. IEEE Access.

[5] Loukas G., Karapistoli E., Panaousis E., Sarigiannidis P., Bezemskij A., Vuong T. (2019). A taxonomy and survey of cyber-physical intrusion detection approaches for vehicles. Ad Hoc Netw..

[6] Young C., Zambreno J., Olufowobi H., Bloom G. (2019). Survey of automotive controller area network intrusion detection systems. IEEE Des. Test.

[7] Avatefipour O., Al-Sumaiti A.S., El-Sherbeeny A.M., Awwad E.M., Elmeligy M.A., Mohamed M.A., Malik H. (2019). An Intelligent Secured Framework for Cyberattack Detection in Electric Vehicles’ CAN Bus Using Machine Learning. IEEE Access.

[8] Kang M.J., Kang J.W. (2016). Intrusion detection system using deep neural network for in-vehicle network security. PLoS ONE.

[9] Yu K.-S., Kim S.-H., Lim D.W., Kim Y.S. (2020). A Multiple Rényi Entropy Based Intrusion Detection System for Connected Vehicles. Entropy.

[10] Choi W., Joo K., Jo H.J., Park M.C., Lee D.H. (2018). VoltageIDS: Low-level communication characteristics for automotive intrusion detection system. IEEE Trans. Inf. Forensics Secur..

[11] Groza B., Murvay P.S. (2018). Efficient intrusion detection with bloom filtering in controller area networks. IEEE Trans. Inf. Forensics Secur..

[12] Müter M., Asaj N. Entropy-based anomaly detection for in-vehicle networks. Proceedings of the 2011 IEEE Intelligent Vehicles Symposium (IV).

[13] Wu W., Huang Y., Kurachi R., Zeng G., Xie G., Li R., Li K. (2018). Sliding window optimized information entropy analysis method for intrusion detection on in-vehicle networks. IEEE Access.

[14] Bandt C., Pompe B. (2002). Permutation entropy: A natural complexity measure for time series. Phys. Rev. Lett..

[15] Hu Y., Li H., Luan T.H., Yang A., Sun L., Wang Z., Wang R. (2018). Detecting stealthy attacks on industrial control systems using a permutation entropy-based method. Future Gener. Comput. Syst..

[16] Zanin M., Gómez-Andrés D., Pulido-Valdeolivas I., Martín-Gonzalo J.A., López-López J., Pascual-Pascual S.I., Rausell E. (2018). Characterizing normal and pathological gait through permutation entropy. Entropy.

[17] Sharma R., Pachori R.B., Acharya U.R. (2015). Application of entropy measures on intrinsic mode functions for the automated identification of focal electroencephalogram signals. Entropy.

[18] Rodríguez-Sotelo J.L., Osorio-Forero A., Jiménez-Rodríguez A., Cuesta-Frau D., Cirugeda-Roldán E., Peluffo D. (2014). Automatic sleep stages classification using EEG entropy features and unsupervised pattern analysis techniques. Entropy.

[19] Rostaghi M., Azami H. (2016). Dispersion entropy: A measure for time-series analysis. IEEE Signal Process. Lett..

[20] Baldini G., Giuliani R., Steri G., Neisse R. Physical layer authentication of Internet of Things wireless devices through permutation and dispersion entropy. Proceedings of the 2017 IEEE Global Internet of Things Summit (GIoTS).

[21] Rostaghi M., Ashory M.R., Azami H. (2019). Application of dispersion entropy to status characterization of rotary machines. J. Sound Vib..

[22] Deng W., Zhang S., Zhao H., Yang X. (2018). A novel fault diagnosis method based on integrating empirical wavelet transform and fuzzy entropy for motor bearing. IEEE Access.

[23] Varma P.R.K., Kumari V.V., Kumar S.S. (2016). Feature selection using relative fuzzy entropy and ant colony optimization applied to real-time intrusion detection system. Procedia Comput. Sci..

[24] Lima C.F.L., Assis F.M., de Souza C.P. A comparative study of use of Shannon, Rényi and Tsallis entropy for attribute selecting in network intrusion detection. Proceedings of the 2011 IEEE International Workshop on Measurements and Networking Proceedings (M&N).

[25] Bereziński P., Jasiul B., Szpyrka M. (2015). An entropy-based network anomaly detection method. Entropy.

[26] Seo E., Song H.M., Kim H.K. GIDS: GAN based intrusion detection system for in-vehicle network. Proceedings of the 2018 IEEE 16th Annual Conference on Privacy, Security and Trust (PST).

[27] Song H.M., Woo J., Kim H.K. (2020). In-vehicle network intrusion detection using deep convolutional neural network. Veh. Commun..

[28] Wenye G. (2020). Shannon and Non-Extensive Entropy. MATLAB Central File Exchange. https://www.mathworks.com/matlabcentral/fileexchange/18133-shannon-and-non-extensive-entropy.

[29] Azami H., Rostaghi M., Abásolo D., Escudero J. (2017). Refined composite multiscale dispersion entropy and its application to biomedical signals. IEEE Trans. Biomed. Eng..

[30] Pincus S.M. (1991). Approximate entropy as a measure of system complexity. Proc. Natl. Acad. Sci. USA.

[31] Fulcher B.D., Jones N.S. (2017). hctsa: A computational framework for automated time-series phenotyping using massive feature extraction. Cell Syst..

[32] Fulcher B.D., Little M.A., Jones N.S. (2013). Highly comparative time-series analysis: The empirical structure of time series and their methods. J. R. Soc. Interface.

[33] Richman J.S., Moorman J.R. (2000). Physiological time-series analysis using approximate entropy and sample entropy. Am. J. Physiol.-Heart Circ. Physiol..

[34] Azami H., Fernández A., Escudero J. (2017). Refined multiscale fuzzy entropy based on standard deviation for biomedical signal analysis. Med. Biol. Eng. Comput..

[35] Chen W., Zhuang J., Yu W., Wang Z. (2009). Measuring complexity using fuzzyen, apen, and sampen. Med. Eng. Phys..

[36] Delgado-Bonal A., Marshak A. (2019). Approximate entropy and sample entropy: A comprehensive tutorial. Entropy.

